# The innate immune IMD pathway is a key regulator of gut microbiome and metabolic homeostasis in the black tiger shrimp (*Penaeus monodon*)

**DOI:** 10.1371/journal.pone.0338796

**Published:** 2025-12-16

**Authors:** Premruethai Supungul, Sureerat Tang, Tanaporn Uengwetwanit, Umaporn Uawisetwathana, Pacharaporn Angthong, Sopacha Arayamethakorn, Phimsucha Bunphimpapha, Panyisa Potibut, Waraporn Jangsutthivorawat, Virak Visudtiphole, Sage Chaiyapechara, Wanilada Rungrassamee

**Affiliations:** 1 Aquatic Molecular Genetics and Biotechnology Research Team, Integrative Aquaculture Biotechnology Research Group, National Center for Genetic Engineering and Biotechnology, National Science and Development Agency, Pathum Thani, Thailand; 2 Advanced Diagnostics and Biomarker Discovery Research Team, Biosensing and Bioprospecting Technology Research Group, National Center for Genetic Engineering and Biotechnology, National Science and Development Agency, Pathum Thani, Thailand; 3 Aquaculture Service Development Research Team, Integrative Aquaculture Biotechnology Research Group, National Center for Genetic Engineering and Biotechnology, National Science and Development Agency, Pathum Thani, Thailand; Uppsala University, SWEDEN

## Abstract

The gut microbiome plays a fundamental role in host health and homeostasis, yet immune mechanisms regulating this relationship remain poorly understood in commercially important invertebrate such as the black tiger shrimp (*Penaeus monodon*). We employed a multiomics approach, combining RNA interference (RNAi) with transcriptomic, metabolomic, and 16S rRNA gene profiling, to investigate how the innate immune Toll and IMD pathways regulate gut health. We systematically suppressed key signaling components, *MyD88* (Toll) and *Relish* (IMD), under non-pathogenic conditions. Knockdown of the IMD pathway transcription factor, *Relish*, triggered a profound and selective response across all measured biological layers. We observed a disproportionately large transcriptomic change, with 1,362 differentially expressed genes (DEGs) in the *Relish* knockdown group compared to only 333 DEGs in the *MyD88* knockdown group. This was accompanied by a targeted alteration in immune effectors, including the upregulation of *lysozyme C-like* (log_2_ fold change = 2.44) and a strong suppression of *penaeidin 5* (log_2_ fold change = −3.62). At the microbial level, while overall community structure remained stable, a selective shift was observed, the abundance of specific Gram-negative genera, particularly *Photobacterium* and *Shewanella*, was significantly reduced, yet *Pseudoalteromonas* were enriched in the *Relish* knockdown group. Metabolomic analysis further revealed that the *Relish*-suppressed shrimp had a distinct metabolic signature, marked by a decrease in bacterial-associated metabolites like *D-alanyl-D-alanine* and an increase in pro-inflammatory markers such as succinic acid and 8-HETE. Our findings showed that in *P. monodon*, the IMD pathway is the primary and central regulator of gut microbiome and metabolic homeostasis. This study provides novel insights into the dynamic interplay between innate immunity and the gut microbiome in a crustacean, identifying the IMD pathway as a promising target for developing strategies to enhance shrimp health and the sustainability of the global aquaculture industry.

## Introduction

Shrimp aquaculture is one of important global aquatic food production with significant growth to meet increasing consumer demands [1]. However, this industry has been facing substantial economic losses due to disease outbreaks, which cause high mortality rates and poor growth performance [2]. Historically, prophylactic antibiotic use was common to control for disease control, but concerns regarding antibiotic residues, environmental contamination, and the escalating threat of antimicrobial resistance have led to widespread bans or strictly regulated used [3–5]. Consequently, shrimp farming practices have shifted towards modulating the gut microbiota to enhance host resilience as one approach to lower disease outbreak incidents. This has driven the search for sustainable alternatives, with functional feed additives like probiotics, prebiotics, and synbiotics emerging as promising means to enhance shrimp growth performance and health [6,7]. A better understanding of the complex relationship between host immunity and the gut microbiome is crucial for the sustainable development of feed additives in shrimp production.

Unlike vertebrates, crustaceans, including the black tiger shrimp (*Penaeus monodon*), rely exclusively on their innate immune system as the primary defense against pathogens [8]. The system consists of both cellular and humoral responses such as phagocytosis, apoptosis, the prophenoloxidase system, and the production of antimicrobial peptides (AMPs) [9–11]. These responses are regulated by the highly conserved Toll and immune deficiency (IMD) signaling pathways [12]. The Toll pathway is primarily involved in defense against Gram-positive bacteria and fungi, while the IMD pathway is crucial for responding to Gram-negative bacteria and viruses, both converging on the induction of AMPs and other immune effectors [13–16]. There are studies reporting how the host shrimp shapes its gut microbial community through a combination of physical barriers and innate immune effectors. A main physical barrier of shrimp is the peritrophic membrane (PM), a semi-permeable chitin-protein layer lining the midgut [17]. The PM not only functions in digestion but also act as a barrier between the microbiota and the intestinal epithelium. Therefore, a disruption of the PM leads to increased susceptibility to pathogens like *Vibrio parahaemolyticus*, the causative agent of Acute Hepatopancreatic Necrosis Disease (AHPND) by allowing pathogen colonization in shrimp intestine [18]. In addition, the shrimp’s antimicrobial peptides (AMPs), such as penaeidin and crustin [19] also play parts in maintaining the balance of shrimp gut microbiome [20].

Beyond responding to pathogen and stress responses, growing evidence from invertebrate organisms indicates the immune pathways are also fundamental to maintaining homeostasis with the resident gut microbiota [21–23]. In *Drosophila*, the IMD pathway is not only important for systemic defense against pathogens but also for actively managing the population of commensal bacteria, a function distinct from its role in inducing antimicrobial peptides [24]. In the red palm weevil (*Rhynchophorus ferrugineus*), silencing the IMD pathway’s key transcription factor, *Relish*, compromised the insect’s ability to clear pathogens and led to a significant increase in the total gut bacterial load, altering the commensal community structure [25]. This demonstrates that host immunity engages in a dynamic crosstalk to shape a stable gut ecosystem. While these studies in insects establish the IMD pathway as a key regulator of the gut ecosystem, a significant knowledge gap remains in commercially important crustaceans. In shrimp, it is largely unknown how the central Toll and IMD immune signaling pathways directly regulate the composition and function of the resident gut microbiota under non-pathogenic, homeostatic conditions.

Here, we aimed to investigate the relationship between host and gut microbiota in the black tiger shrimp (*P. monodon*), specifically how the dynamics of the gut microbiota changed when host immune pathways was disrupted. To address this, we employed an integrated multi-omics approach to dissect the roles of the Toll and IMD pathways in governing gut homeostasis in *P. monodon*. Using RNA interference (RNAi), an important tool for elucidating immune mechanisms in shrimp, the key signaling components *MyD88* (Toll pathway) and *Relish* (IMD pathway) of *P. monodon* were suppressed. By combining this immune perturbation using RNAi with 16S rRNA gene sequencing, host transcriptomics (RNAseq), and metabolomics, our main objectives were to validate the specific knockdown of *MyD88* and *Relish* and determine their impact on host-gut microbial homeostasis. Our findings provided novel insights into the dynamic interplay between the innate immune system and resident bacteria in the shrimp gut, establishing a foundation for developing shrimp health management strategies in sustainable aquaculture.

## Materials and methods

### Ethics statement

All experimental protocols were reviewed and approved by the Animal Ethics Committee at the National Center for Genetic Engineering and Biotechnology (approval code BT-Animal 04/2560) and were conducted in accordance with relevant institutional guidelines and national regulations. Following the experiment, the shrimp were humanely euthanized using a 1-step rapid chilling approach (2°C to 4°C) to induce immediate anesthesia and ensure death, in compliance with the AVMA Guidelines for the Euthanasia of Animals [26].

### Shrimp experimental trial

Black tiger shrimp postlarvae were obtained from the Shrimp Genetic Improvement Center, National Center for Genetic Engineering and Biotechnology (BIOTEC, Surat Thani, Thailand). The shrimp population was tested to be free of specific pathogens (Taura Syndrome Virus, White Spot Syndrome Virus, Yellow Head Virus, and Infectious Hypodermal and Hematopoietic Necrosis Virus) using EZEE GENE PCR test kits (BIOTEC, Thailand). Shrimp were acclimated in a 2,000 L tank in a recirculating system at the Aquaculture Service Development facility (BIOTEC, Pathum Thani, Thailand). They were fed with commercial feed pellets (Inteqc, Thailand) at approximately 5% of body weight per day for five meals. Water quality was monitored every other day for temperature, pH, and dissolved oxygen and weekly for ammonia-nitrogen, nitrite-nitrogen, and alkalinity levels. Shrimp weighing 3–5 g (approximately 3-month-old shrimp) were used for the RNAi knockdown experiment.

### Construction of dsRNA plasmids and bacterial expression of dsRNAs for RNAi knockout of the innate immune response pathway

To develop the double stranded RNA (dsRNA) for studying the microbial population in the intestine of host shrimp, dsRNA was synthesized using *Escherichia coli* as the host [27]. The fragment of MyD88 or Relish gene (including the inverted direction of the gene) and the fragment of *gfp* were amplified. Each DNA fragment (MyD88 or Relish gene, GFP and inverted MyD88 or Relish gene) was ligated into pET17b. The plasmids were used for *in vitro* production of double-stranded MyD RNA and double-stranded Relish RNA. The plasmids were transformed into *E. coli* HT115, an RNase III deficient strain, by the heat shock method. The overnight bacterial culture was diluted 100-fold with 2XYT media containing ampicillin (100 μg/ml) and tetracycline (12.5 μg/ml) and grown at 37°C until the OD600 reached 0.4. Then, 0.4 mM IPTG was added for 4 h. Cell pellets were harvested by centrifugation at 6000xg for 5 min at 4°C.

To extract dsRNA from the bacteria, the harvested cells were fixed in 75% ethanol for 5 min. The fixed cell was collected by centrifugation and then resuspended in 150 mM NaCl and incubated at room temperature for 1 h. The dsRNA was treated with RNase A to remove single stranded RNA for 15 min at 37°C and was extracted with 10 mL Trizol, dissolved in 150 mM NaCl and stored at −80°C until use.

### RNAi-mediated suppression of the *Pm*MyD88 and *Pm*Relish

**Fig 1:**
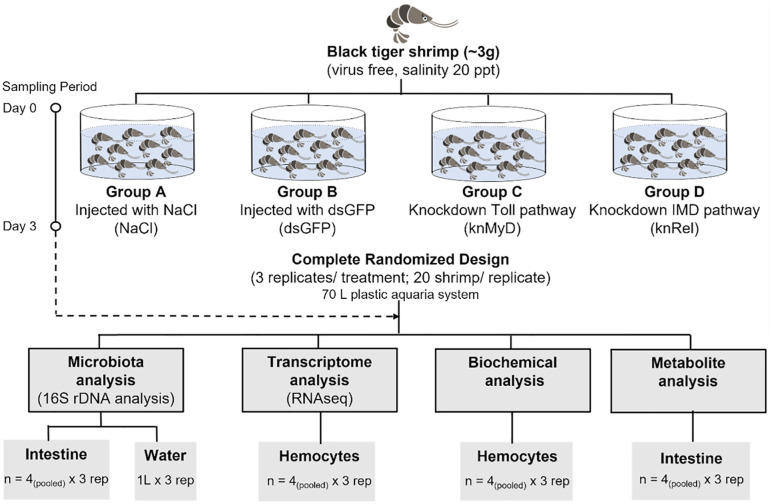
Schematic representation of the experimental workflow to investigate the impact of RNAi-induced immunosuppression on gut microbiota composition in the black tiger shrimp (*P. monodon*). Four treatment groups in this study were Group A (NaCl), the negative control group injected with saline solution, and Group B (dsGFP), the negative control group for RNAi treatment, where shrimp were injected with dsRNA targeting GFP, a gene not found in shrimp. Group C (knMyD) was the experimental shrimp injected with dsRNA to silence the *PmMyD88*, a key component of the Toll immune pathway, and Group D (knRel) was another experimental group of shrimp juveniles, injected with dsRNA to silence the Relish gene, a key component of the IMD immune pathway. Tissue samples were collected after three days of the treatments for multi-omics analysis.

Shrimp were divided into four groups (Fig 1). Double-stranded RNA targeting *PmMyd88* (ds*Pm*MyD88 RNA), *PmRelish* (ds*Pm*Relish RNA) or *GFP* (dsGFP RNA) as a negative control, was prepared. Each dsRNA (37.5 μg equivalent to 7.5 μg per 1 g shrimp) was dissolved in 30 μL of 150 mM NaCl and injected into the lateral region of the fourth abdominal segment. An additional control group was injected with 150 mM NaCl only. The injection of dsRNA (7.5 μg per 1 g shrimp) and NaCl was repeated at 24 h after the first injection. Hemolymph was collected from twenty-four individual shrimp per group and pooled (4 shrimp/sample) for hemocyte collection, total RNA extraction and first strand cDNA synthesis using our previously described methods [28,29]. The suppression of the target genes was subject to be validated by quantitative realtime RT-PCR (qRT-PCR). The efficiency of suppression was calculated as the percentage reduction in transcript level, defined as: $\text{1}\text{0}\text{0}\%-\left[\frac{\left(\text{Transcript}\;\text{level}\;\text{in}\;\text{knMyD88}\;\text{or}\;\text{knRel}\right)}{\left(\text{Transcript}\;\text{level}\;\text{in}\;\text{dsGFP}\;\text{control}\right)}\;\text{x}\;\text{1}\text{0}\text{0}\%\right]$. Shrimp tissues were collected for microbiome, transcriptome and metabolomics analyses (Fig 1).

### Validation of RNAi-mediated suppression by quantitative realtime PCR analysis

The qRT-PCR was performed in LightCycler^®^ 480II (Roche) using Luna Universal qPCR Master Mix (Biolabs) with specific forward and reverse primers for the *Pm*MyD88 and *Pm*Relish genes (S1 Table). The housekeeping gene, *elongation factor 1a* (*EF1a*) was used as an internal control. Cycling parameters were an initial activation at 95°C for 1 min followed by 40 cycles of 95°C for 15 s and 60°C for 30 s. The fluorescent signal intensities were recorded at the end of each cycle. Melting curve analysis was performed from 60 to 95°C with continuous fluorescent reading every 0.5°C increments to confirm that only one product was amplified. Relatively to the expression of EF1a transcripts, the relative expression was calculated by the 2^-ΔΔct^ method [30]. The statistical significance of any differences was tested by using a one-way ANOVA followed by the Duncan’s new multiple range test using the SPSS software. Significance was accepted at the *p *< 0.05 level.

### Analyses of total and differential hemocyte counts

Hemocytes were fixed by adding 10% formalin in 0.45 M NaCl to hemolymph in a 1:1 ratio for 10 min at room temperature. Twenty µL of the sample was then incubated with 20 µL of 1.2% Rose Bengal (RB) in 50% ethanol at room temperature for 20 min. The total number of hemocytes (THC) was determined under the microscope using a hemocytometer.

For differential hemocyte count analysis (DHC), the sample stained with RB was smeared on a slide and air-dried. The slide was then stained with hematoxylin for 10 min and rinsed with water. The slide was then immersed 10 times in 70% ethanol followed by 10 times in 100% ethanol. The slide was dipped in xylene 3 times for 3 min each before being coated with permount solution and covered with a coverslip. Hemocyte types were differentiated under the microscope from a total of 200 hemocytes. The proportion of each cell type (% Cells) was calculated as the ratio of the differential cell count divided by the total hemocyte count (THC). Both THC and DHC were performed using duplicate slides for each sample.

### Construction of DNA libraries for next-generation sequencing analysis

DNA samples were extracted from each shrimp intestine using a QIAamp DNA extraction kit (Qiagen) according to the manufacturer’s instruction. All genomic DNA samples were further purified using the genomic DNA clean & concentrator™ (Zymo Research, USA). DNA concentration was determined using a Nanodrop ND-8000 spectrophotometer (Thermo Fisher Scientific, USA). The V3 and V4 variable regions of the 16S rRNA genes were amplified by the primer pair; 16S_F (5’TCGTCGGCAGCGTCAGATGTGTATAAGAGACAGCCTACGGGNGGCWGCAG 3’) and 16S_R (5’GTCTCGTGGGCTCGGAGATGTGTATAAGAGACAGGACTACHVGGGTATCTAATCC 3’) for next‐generation sequencing‐based diversity analyses, in which Illumina adapter overhang nucleotide sequences were added to the gene‐specific sequences. An amount of 100 ng of DNA sample was used as PCR template. Each DNA library for intestinal bacterial communities was amplified with the primer pair 16S_F and 16S_R using Q5 high-fidelity DNA polymerase (New England Biolabs Inc., USA). The PCR cycle parameters were an initial denaturation at a 94°C for 3 min, followed by 25 cycles of 94°C denaturation for 30 s, a 57°C annealing for 45 s, and a 72°C extension for 45 s, and a final extension at 72°C for 5 min. The cycles were replicated to minimize saturation bias, and the reactions were set in duplicate. Each replicate was pooled together. The PCR products were purified with a Qiaquick gel extraction kit (Qiagen, USA). DNA concentration was measured with a Nanodrop ND-8000 spectrophotometer and visualized by agarose gel electrophoresis. All amplicons were in the expected size of the single band and showed high purity. Total genomic DNA from each library was adjusted to 10 ng/μL in a volume of 40 μL and subjected to 16S rRNA amplicon sequencing using Illumina MiSeq next-generation sequencing service (Macrogen Inc, Korea). Each DNA library (n = 30) was sequenced twice for technical replicates.

### Microbiota data analysis

Raw paired-end sequences of 16S rRNA were processed with Trimgalore [31] to remove adapter overhangs and low-quality bases (Phread score <20). Trimmed reads were imported into QIIME2 (release version 2020.8) [32]. Sequences were demultiplexed using DADA2 to generate amplicon sequence variants (ASVs) [33]. The taxonomy was assigned using the SILVA database (version 132) with BLAST+ consensus taxonomy classifier. ASVs were aligned using MAFFT [34] and then phylogeny was generated using FastTree [35]. Data analysis was performed using R 3.5.2 (RStudio Team, 2015, R Core Team, 2018). Alpha and beta diversity analyses were performed using the MicrobiomeAnalyst [36]. Linear discriminant analysis effect size (LEfSe) was used to determine statistical significance of taxon abundance between treatment and control groups [37]. The LEfSe cutoff for significantly different abundant taxa was the p-value < 0.05 in the Kruskal-Wallis test and Wilcoxon test, and a threshold for the logarithmic linear discriminant analysis (LDA) score > 2.0. The pattern of bacterial community co-occurrence in each group was constructed using Co-occurrence Network Inference (CoNet) [38] under Cytoscape v3.7 [39]. Association between bacteria was assessed by calculating the Spearman correlation coefficient (threshold 0.7).

### Transcriptomic analysis of shrimp intestine

Total RNA from shrimp tissue sample was extracted using TRI Reagent (Molecular Research Center, USA) according to the manufacturer’s protocol. Each hemocyte sample was ground with a sterile pestle in TRI reagent and subjected to chloroform extraction. All RNA samples were treated with 0.5 units/µg RQ1 RNase-free DNase (Promega, USA) to remove potential DNA contamination. RNA purity and concentration were analyzed by NanoDrop (ND-8000) spectrophotometer and visualized by 1% agarose gel electrophoresis. Each RNA library was prepared using a TruSeq Stranded mRNA LT Sample Prep kit (Illumina, USA) according to the manufacturer’s protocol. RNA sequencing analysis was performed using the Illumina Hiseq platform (Illumina, USA) (Macrogen Inc., Korea).

Low-quality bases (quality score < 20) and adapter sequences were removed from both ends using Trimmomatic v.0.32 [40]. The clean reads were mapped to the *P. monodon* genome GCF015228065.1 [41] using STAR [42]. Gene count matrices were generated using FeatureCounts [43]. Counts were normalized and differential gene expression analysis was performed using DESeq2 [44].

Genes with log_2_ (foldchange) ≥ 1 and a p-value < 0.05 were considered as differentially expressed genes (DEGs).

### Untargeted metabolite profiles analysis in shrimp intestines using LC-MS/MS

Shrimp intestine samples (~ 100 mg fresh weight per sample) were prepared and analyzed according to a previous study [45]. Briefly, each frozen tissue was ground to a fine powder and extracted with 1 mL of cold ethyl acetate:acetone (15:2 v/v). The supernatant was subsequently collected and dried under vacuum at 35°C for 20 min. The dried pellet was reconstituted with 150 µL of acetonitrile (Optima LC/MS grade). A flow-through sample (100 µL) was transferred to an LC vial with a micro-insert before injection into LC-HRMS. Pooled biological samples (n_pooled_ = 34) were used as quality control samples and injected after every 10 samples to check the reproducibility of the instrument. A blank sample, the extract without shrimp intestine, was also run in parallel to subtract any background signals.

Untargeted metabolite profiles were generated using the Dionex RS3000 in conjunction with a Thermo Scientific™ Orbitrap Fusion™ Tribrid™ mass spectrometer. Reverse-phase chromatography was performed with an HSS T3 C18 column (2.1 x 100 mm, 1.8 µm, Thermo) using a mobile phase (solvent A: water + 0.1% acetic acid and solvent B: acetronitrile + 0.1% acetic acid) with a gradient elution system as follows: an isocratic period for 2 min at 99:1 (A:B), followed by a linear gradient to 1:99 (A:B) for 18 min and a hold for 5 min, followed by restoration of initial conditions 99:1 (A:B) for 1 min and a hold for 5 min. Mass spectra were acquired using the data-dependent full scan MS/MS under negative electrospray ionization (ESI) mode over the mass range 50–1200 Da. The ion source was operated at a voltage of 3,500 V, an ion transfer temperature of 325°C, and a vaporizer temperature of 275°C. The MS full scan resolution was set to 120,000 FWHM and the MS2 scan resolution was set to 15,000 FWHM with 25% higher-energy collisional dissociations (HCD).

### Metabolite data processing and analyses

The acquired MS data from the samples, blanks and pooled samples were processed using Compound Discoverer (CD) 3.0 software with a workflow as follows: retention time alignment, unknown compound detection, elemental composition prediction, chemical background subtraction using blanks and compound identification using ChemSpider [46], HMDB [47], KEGG [48] LipidMAPS (formula or exact mass, www.lipidmaps.org/) and mzCloud (mass fragmentation patterns, www.mzcloud.org). The metabolite features annotated according to aforementioned criteria were collected for further analysis. Partial least square-discriminant analysis (PLS-DA) was used to classify among the treatment groups using SIMCA 16 (Umea, Sweden). Variable importance in projection (VIP) with a value in negative ESI mode greater than one was selected. The selected metabolite features were then analyzed between the Toll (MyD)-suppressed and IMD (Relish)-suppressed groups compared with the control (dsGFP). Metabolites features with a greater than 2.0-fold change (immune-suppressed/control groups) and annotation confidence greater than 90% were selected for clustergram visualization using Cluster 3.0 software [49].

## Results

### Validation of RNAi-mediated suppression of the MyD88 and Relish in the black tiger shrimp

**Fig 2:**
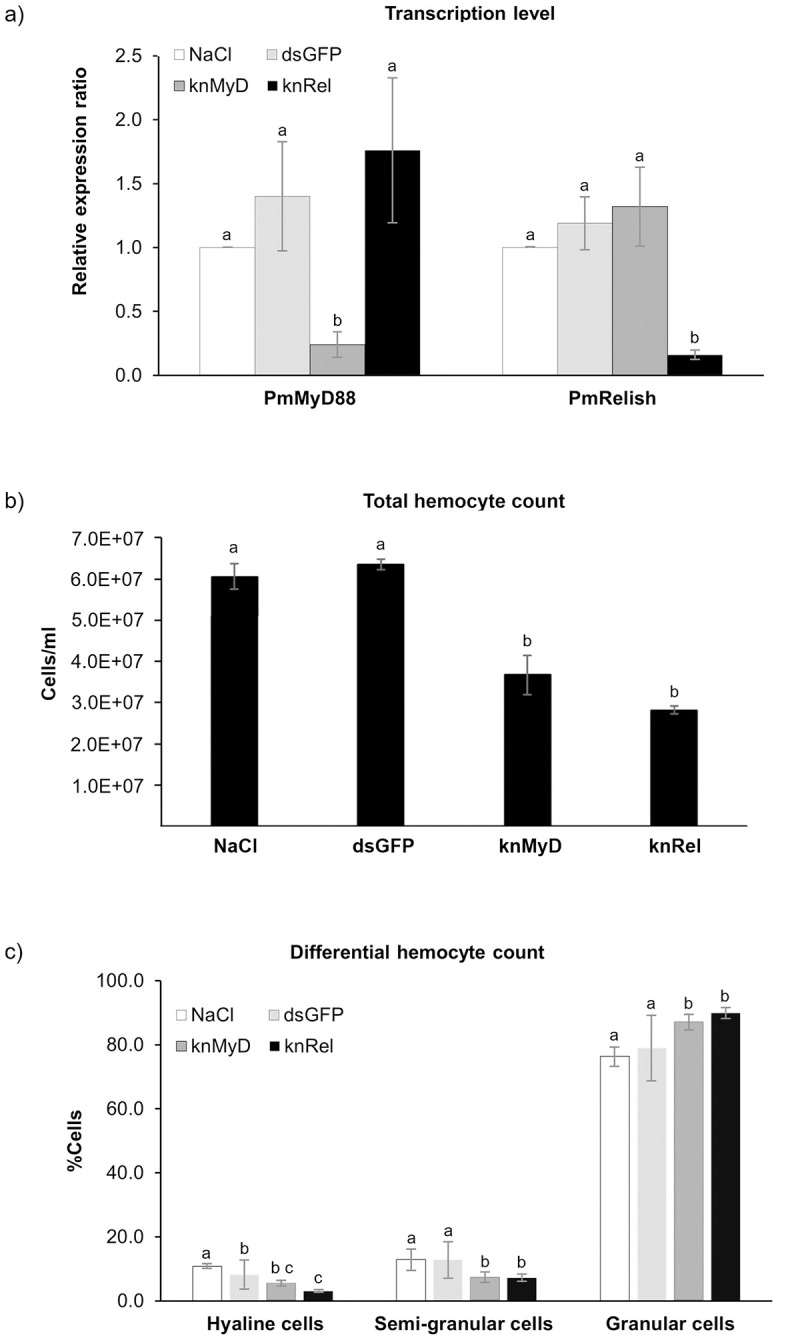
Effects on gene expression and hemocyte populations following RNAi-mediated knockdown of *PmMyD88* (knMyd88) and *PmRelish* (knRel) in *P. monodon.* **(a)** Gene expression analysis of *PmMyD88* and *PmRelish* transcript levels in hemocytes following RNAi treatment. The dsGFP injected shrimp group served as a negative control. **(b)** Total hemocyte counts are represented as the number of hemocyte cells per mL of hemolymph. **(c)** Differential hemocyte counts are represented as the percentage of different cell types relative to the total hemocyte count. Significant differences (*p* < 0.05) are denoted by different letters.

Here, the specific and effective knockdown of *PmMyD88* and *PmRelish* in the hemolymph of *P. monodon* were confirmed via qRT-PCR and the effects of RNAi-mediated suppression of *PmMyD88* and *PmRelish* on hemocyte homeostasis (Fig 2). Injection of double-stranded RNA targeting *Pm*MyD88 resulted in a significant and specific reduction in *PmMyD88* transcript levels without affecting *PmRelish* expression (Fig 2a). *PmMyd88* transcript levels were suppressed by 83% on Day 3 (48 h after the second injection). Conversely, the double-stranded RNA targeting *Pm*Relish also specifically suppressed the transcription level of *PmRelish* but not *PmMyD88* (Fig 2a), showing an efficacy of 87% suppression at the Day 3 timepoint. These results clearly demonstrated the high specificity and sustained efficacy of the RNAi-mediated gene silencing throughout the experimental period. We then investigated the effects of this RNAi-mediated suppression on hemocyte homeostasis. The total and differential hemocyte counts were quantified in shrimp. Knockdown of *PmMyD88* and *PmRelish* led to a significant decrease (*p* < 0.05) in the total hemocyte count (THC) (Fig 2b). Further analysis of the differential hemocyte counts (DHC) revealed a shift in relative proportions of the total cell counts (Fig 2c). In the control groups (NaCl and dsGFP), granular cells were the dominant population, representing approximately 75–80% of total hemocytes. However, following knockdown of *PmMyD88* (knMyD) and *PmRelish* (knRel), the proportions of hyaline and semi-granular cells were significantly reduced (*p *< 0.05), while the proportion of granular cells significantly increased to approximately 88–90%. This showed that the total hemocytes (including all types) was compromised by immune suppression, and the granular cell population became proportionally dominant in the total hemocyte pool.

### Differential gene expression analysis in shrimp hemocytes under RNAi knockdown of *Pm*MyD88 (knMyD) to the control and RNAi knockdown of *Pm*Relish (knRel) to the control

To investigate the impact of suppressing the Toll and IMD pathways on the host immune system, we profiled gene expression in shrimp hemocytes using RNA sequencing. RNAi-mediated knockdown of the key transcription factor *PmRelish* (knRel) from the IMD pathway resulted in a substantial transcriptomic response, with a total of 1,362 differentially expressed genes (DEGs) identified. In contrast, knockdown of the Toll pathway adaptor protein *PmMyD88* (knMyD) resulted in a much more modest response, with only 333 DEGs identified (S1 Fig and S2 Table). Our transcriptomic data also confirmed the specific and effective suppression of our target genes in which *PmMyD88* was significantly downregulated in the knMyD group (log_2_ fold change = −1.46), and PmRelish was significantly downregulated in the knRel group (log_2_ fold change = −2.09; Table 1). Interestingly, the knockdown of *PmRelish* also affected the Toll pathway, with several components, including the *toll-like receptor* (log_2_ fold change = −1.74) and *spaetzle 3-like protein* (log_2_ fold change = −1.13), showing significant downregulation. Conversely, we observed a shared response in both knockdown groups, characterized by the upregulation of several ankyrin repeat-containing proteins (e.g., *ankyrin repeat and SOCS box protein 10-like*; Table 1). These proteins are involved in regulating the IMD pathway, suggesting a complex feedback loop or compensatory mechanism.

**Table 1:** Differentially expressed immune-related genes in shrimp hemocytes. Differential expression of immune-related genes following RNAi-mediated knockdown of MyD88 (knMyD) or Relish (knRel) relative to a dsGFP-injected control. Values are presented as the log_2_ fold change. Bold values indicate statistically significant changes, defined as an absolute log_2_ fold change ≥1 and a p-value < 0.05.

Gene description	Log_2_ (knMyD/dsGFP)	Log_2_ (knRel/dsGFP)
**IMD pathway**		
*NF-κB protein (Relish)*	−0.03	**−2.09**
*ankyrin repeat and SOCS box protein 10-like*	**1.42**	**1.81**
*ankyrin repeat domain-containing protein 1-like*	**1.27**	**1.72**
*ankyrin-1-like*	**1.28**	**1.71**
*ankyrin-2-like*	0.08	**1.42**
*E3 ubiquitin-protein ligase arih1-like*	0.00	**1.34**
*serine/threonine-protein phosphatase 6 regulatory ankyrin repeat subunit A-like*	**−4.50**	**−4.88**
*serine/threonine-protein phosphatase 6 regulatory ankyrin repeat subunit B-like*	1.32	**1.87**
*E3 ubiquitin-protein ligase SMURF1-like isoform X1*	−0.24	**−1.08**
*E3 ubiquitin-protein ligase SMURF1-like isoform X3*	−0.24	**−1.08**
**Toll pathway**		
*MyD88*	**−1.46**	−0.10
*toll-like receptor Tollo*	−0.03	**−1.74**
*spaetzle 3-like*	−0.19	**−1.13**
*ras-related protein Rab-44-like*	0.36	**1.39**
*ras-related protein Rap-1b-like*	−0.76	**−1.49**
**Antimicrobial peptide**		
*anti-lipopolysaccharide factor 9*	0.28	**1.53**
*anti-lipopolysaccharide factor isoform 2*	0.19	**1.34**
*anti-lipopolysaccharide factor-like*	−0.45	**1.31**
*anti-lipopolysaccharide factor-like isoform X1*	−0.40	**1.20**
*epsin-like*	**−1.87**	**−1.53**
*lysozyme C*	0.11	**1.78**
*lysozyme C-like*	0.80	**2.44**
*lysozyme C-like isoform X2*	0.30	**2.27**
*penaeidin-2b-like*	−0.25	**−1.38**
*penaeidin 5 antimicrobial peptide*	−0.69	**−3.62**
*penaeidin-4c-like*	−0.13	**−1.57**
*stylicin 1*	−0.31	**−2.06**
**Apoptosis**		
*CASP8 and FADD-like apoptosis regulator isoform X1*	0.78	**1.19**
*caspase-2-like isoform X2*	0.78	**1.21**
*caspase-8-like*	0.07	**1.07**
*hemocyte homeostasis-associated protein*	**1.70**	**3.37**
*programmed cell death protein 4-like*	−0.19	**−1.33**
**Heat shock protein and chaperone**		
*BAG family molecular chaperone regulator 1-like isoform X1*	**1.11**	**2.12**
*dnaJ homolog shv-like*	0.05	**1.03**
*endoplasmic reticulum chaperone BiP*	0.00	**1.25**
*heat shock 70 kDa protein cognate 4-like*	0.10	**1.55**
*heat shock protein*	1.33	**2.81**
*heat shock protein HSP 90-alpha-like*	0.71	**2.23**
*Hsp90*	0.65	**2.22**
*peptidyl-prolyl cis-trans isomerase B1-like*	0.92	**−2.06**
*sacsin-like*	0.67	**1.21**
*tubulin-specific chaperone cofactor E-like protein*	−0.19	**−1.32**
**ProPO system**		
*hepatocyte growth factor activator-like isoform X1*	−0.77	**−2.41**
*masquerade-like serine proteinase-like protein 3*	−0.28	**−1.64**
*brachyurin-like*	**−4.06**	−1.88
*dihydroorotate dehydrogenase (quinone)*	0.91	**1.36**
*hemocyanin F chain-like*	−0.10	**1.05**
*prostaglandin reductase 1-like*	**−1.29**	−0.74
*putative dihydroorotate dehydrogenase (quinone)*	−0.91	**1.94**
**Protease and protease inhibitor**		
*alpha-1-macroglobulin-like*	**1.34**	1.16
*alpha-2-macroglobulin-like*	1.43	**2.06**
*cathepsin L-like*	0.48	**1.24**
*chymotrypsin-like protease CTRL-1*	**−5.67**	**−4.30**
*clotting factor B-like*	−0.21	**1.61**
*kunitz-type serine protease inhibitor HCRG2-like*	0.03	**1.89**
*metalloprotease TIKI2-like*	−0.70	**−1.34**
*presequence protease, mitochondrial-like*	0.59	**1.07**
*serine protease inhibitor 88Ea-like*	**−2.88**	**−1.70**
*tetraspanin-1-like*	**−2.02**	−1.11
*trypsin beta-like*	**2.50**	1.79
*protease-like isoform X2*	**−1.16**	0.49
**Recognition**		
*astakine precursor*	**−2.84**	**−2.96**
*astakine isoform X1*	**−2.84**	**−2.96**
*calreticulin-like*	0.20	**1.18**
*CD209 antigen-like protein A*	−0.19	**2.01**
*coagulation factor IX-like*	−0.46	**−1.41**
*C-type lectin domain family 10 member A-like*	0.82	**1.76**
*C-type lectin domain family 4 member D-like*	−0.05	**2.14**
*cytokine receptor-like*	−0.07	**4.50**
*epidermal growth factor receptor-like*	−0.03	**−1.63**
*galactose-specific lectin nattectin-like*	0.25	**1.10**
*galectin-9-like*	0.69	**1.03**
*laminin subunit gamma-1-like*	−0.50	**−1.31**
*multiple epidermal growth factor-like domains protein 6*	**6.28**	**3.42**
*perlucin-like protein*	**−1.34**	−0.87
*protein dhs-3-like*	**−2.22**	**−1.51**
*protein regulator of cytokinesis 1-like*	**1.05**	**1.09**
*putative tyrosine-protein phosphatase non-receptor type 4 isoform X4*	1.05	**1.43**
*suppressor of cytokine signalling-2 like protein*	0.40	**1.72**
*tachylectin-5A-like*	0.40	**1.32**
**Others**		
*dual oxidase maturation factor 1-like (DUOXA1)*	6.05	**7.56**
*interferon alpha-inducible protein 27-like protein 2*	**3.82**	**3.87**
*mucin-5 AC-like*	0.63	**1.40**
*chitin deacetylase 7-like*	0.09	**2.19**
*crustacyanin-A2 subunit-like*	**−0.90**	**4.46**
*mucin-2-like, partial*	**2.18**	**2.07**
*peroxiredoxin*	−0.18	**1.45**

Suppression of the Toll and IMD pathways resulted in distinct changes across several key immune-related functional categories, particularly within antimicrobial peptides (AMPs). In the knRel group, a selective response characterized by the significant upregulation of several isoforms of *anti-lipopolysaccharide factor* (*ALF*) and *lysozyme C* (including *lysozyme C-like* variant with a log_2_ fold change of 2.44). In contrast, transcripts for various *penaeidins* and *stylicin 1* were strongly decreased (e.g., *penaeidin 5* with a log_2_ fold change of −3.62). This suggests a targeted and differential regulation of immune-related genes. Interestingly, the *epsin-like* transcript was the only AMP suppressed in both the knMyD (log_2_ fold change = −1.87) and knRel (log_2_ fold change = −1.53) groups, suggesting its regulation might be controlled by a different or shared upstream mechanism. Beyond AMPs, we observed significant changes in other functional categories (Table 1). Genes associated with apoptosis and stress response, such as the *hemocyte homeostasis-associated protein* (upregulated 3.37-fold in the knRel group) and most heat shock proteins (e.g., log_2_ fold change of 2.81), were significantly upregulated, particularly in the knRel group. This showed that suppressing the IMD pathway induced a broader stress and pro-survival response in hemocytes. In contrast, key components of the prophenoloxidase (proPO) system, a central part of shrimp immunity, were downregulated in the knRel group. We also noted a profound and shared downregulation of the *chymotrypsin-like protease CTRL-1* in both the knMyD (log_2_ fold change = −5.67) and knRel (log_2_ fold change = −4.30) groups, suggesting a potential convergence point for these two pathways. Furthermore, the knockdown of *PmRelish* and *PmMyD88* had distinct effects on genes involved in pathogen recognition and immune signaling (Table 1). The knRel group showed a notable upregulation of genes like *CD209 antigen-like protein A* (log_2_ fold change = 2.01) and *C-type lectin domain family 4 member D-like* (log_2_ fold change = 2.14). Conversely, the transcript for *multiple epidermal growth factor-like domains protein 6* was highly upregulated in the knMyD group (log_2_ fold change = 6.28), while a *cytokine receptor-like* transcript was highly upregulated in the knRel group (log_2_ fold change = 4.50). Conversely, the *astakine precursor*, a key cytokine in shrimp immunity, was strongly suppressed in both groups (log_2_ fold change ≈ −2.9), suggesting a common regulatory node for this signaling molecule. In addition to these functional categories, we identified several other transcripts with significant and notable changes, suggesting broader compensatory responses to immune stress. For instance, the transcript for *dual oxidase maturation factor 1-like* (DUOXA1) was highly upregulated in both knockdown groups but significantly expressed in the knRel group (log_2_ fold change = 7.56). This gene is known to be involved in maintaining gut barriers in other invertebrates, which could represent a host’s attempt to compensate for a compromised systemic immune response. Similarly, *interferon alpha-inducible protein 27-like protein 2*, a conserved host response to immune stress, was strongly upregulated in both the knMyD and knRel groups (log_2_ fold change = 3.82 and 3.87, respectively). The significant upregulation of the *crustacyanin-A2 subunit-like* gene (log_2_ fold change = 4.46) in the knRel group is also noteworthy, as crustacyanins are a family of proteins that act as immune effectors. This upregulation further suggested the involvement of the IMD pathway for a broad spectrum of immune-related functions.

To validate the transcriptomic profiling, expression levels of selected immune-related transcripts were analyzed by real-time PCR. Comparison of differential expression of genes by RNAseq and real-time PCR techniques showed a correlation between real-time PCR and RNAseq (S2 Fig).

### Microbiome sequencing depth and intestinal microbiota profiles

**Fig 3:**
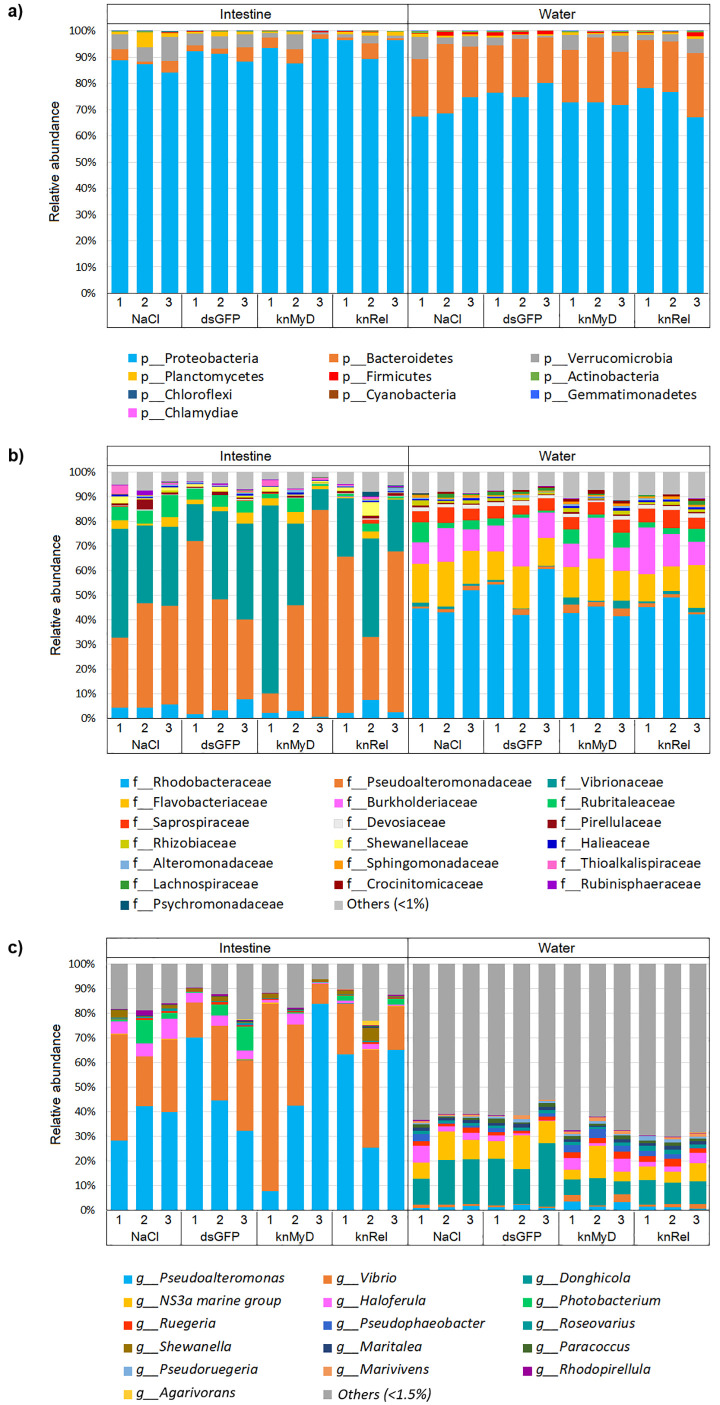
Relative abundance of microbial communities in shrimp intestines and rearing water. Stacked bar plots comparing the relative abundance of microbial community compositions in the intestine and rearing water of black tiger shrimp (*P. monodon*) under different treatment conditions. The experimental groups include two controls, shrimp injected with a saline solution (NaCl) and shrimp injected with double-stranded green fluorescent protein (dsGFP), and two experimental groups, shrimp with RNAi-mediated knockdown of the *MyD88* gene (knMyD) and shrimp with knockdown of the *Relish* gene (knRel) (n = 3 in each group). **a)** Microbial community composition at the phylum level. **b)** microbial community composition at the family level, with “Others (<1%)” representing families with less than 1% relative abundance, **c)** microbial community composition at the genus level, with “Others (<1.5%)” representing genera with less than 1.5% relative abundance.

The microbial profiles in the intestines and rearing water of the four groups were analyzed as follows: *i)* shrimp with RNAi knockdown at MyD88 (knMyD), *ii)* RNAi knockdown via Relish (KnRel), *iii)* a shrimp injected with blank saline solution (NaCl) as a control and *iv)* shrimp injected with double stranded GFP (dsGFP) as a control of RNAi treatment by 16S amplicon sequencing analysis. Rarefaction curves for all samples reached a plateau (S3 Fig), indicating that the sequencing depth reached a saturation level. The relative abundance of bacterial communities associated with the intestines of shrimp in all four treatment groups, and their rearing water was determined (Fig 3). Overall, the relative abundance of bacteria in intestines was similar in the four treatment groups, with the major phyla being Proteobacteria, Bacteroidetes, Verrucomicrobia, and Planctomycetes (Fig 3a), whereas the relative abundance of bacteria in the rearing water showed a different composition than in the shrimp intestines. The major bacterial phyla in all rearing water samples were dominated by Proteobacteria, Bacteroidetes, Verrucomicrobia and Firmicutes during cultivation. However, the relative abundance in both intestines and rearing water at the family level differed slightly among these groups. At the family level, Pseudoalteromonadaceae and Vibrionaceae were predominant in intestines in all groups (Fig 3b). Differences in the intestinal microbiota of shrimp were observed at the genus level. The five most abundant genera in the NaCl and dsGFP groups were *Pseudoalteromonas*, *Vibrio*, *Photobacterium*, *Haloferula,* and *Shewanella*, while *Pseudoalteromonas* was the most abundant genus in the intestines of all treatment groups (Fig 3c). On the other hand, some genera were found in lower abundance in the intestine of shrimp with RNAi knockdown of *PmMyD88* or *PmRelish* signaling pathway. For example, *Haloferula* was present in low abundant in the knMyD group and *Photobacterium* was found in lower abundant or absent in the knRel group.

To further assess the impact of immune suppression on the microbial community structure, we analyzed alpha- and beta-diversity (S4 Fig). Alpha-diversity, measured by the Shannon Index, showed no significant difference among the four treatment groups (NaCl, dsGFP, knMyD, and knRel) within the intestine (*p* = 0.75) or within the rearing water (*p* = 0.14) (S4a Fig). This suggested that the overall community richness and evenness remained stable despite the gene knockdowns. Moreover, beta-diversity was assessed using Principal Coordinates Analysis (PCoA) on weighted UniFrac distances. The PCoA plot clearly demonstrated a strong separation between the intestinal microbiota and the rearing water microbiota (PERMANOVA *p* = 0.001) (S4b Fig), confirming that the shrimp maintain a distinct, gut-specific community. Crucially, the PCoA showed no significant clustering or differentiation among the four treatment groups when analyzing samples exclusively from the intestine (PERMANOVA *p* = 0.18) or exclusively from the water (PERMANOVA *p *= 0.37), which was consistent with the alpha-diversity analysis. Therefore, our alpha and beta diversity analyses showed that the structural stability of the overall gut microbial community was maintained, even as the abundance of some specific genera shifted.

### Comparison of bacterial abundance and co-occurrence network in shrimp intestines under RNAi knockdown of immune pathway

**Fig 4:**
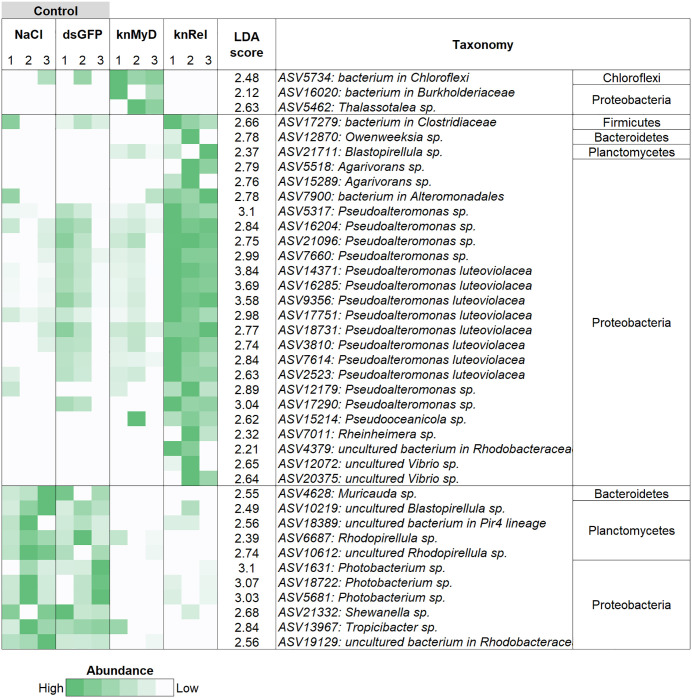
Differential abundance of intestinal bacteria following RNAi knockdown. LEfSe analysis identifying the significantly abundant ASVs in the intestines of *P. monodon* across treatment groups: NaCl, dsGFP, dsMyD88 RNA (knMyD), and dsRelish RNA (knRel).

To determine the shift of bacterial abundance in shrimp intestines under RNAi knockdown of the immune pathway, LEfSe analysis, an algorithm to identify statistically significant bacteria among treatments, revealed some important bacterial changes under RNAi knockdown at *PmMyD88* (knMyD group) or *PmRelish* (KnRel group) (Fig 4). For example, an unclassified bacterial member of Chloroflexi (ASV5734), an unclassifiable member in *Burkholderiaceae* (ASV16020) and *Thalassotalea* sp. (ASV5462) were significantly enriched in the knMyD group. Several members of Firmicutes, Bacteroidetes, Planctomycetes and Proteobacteria were found in significantly higher abundance in the knRel group. Bacterial members belonging to the Bacteroidetes, including *Muricauda* sp. (ASV4628), Planctomycetes such as *Rhodopirellula* sp. (ASV6687), and Proteobacteria such as *Photobacterium* spp. (ASV1631, ASV18722 and ASV5681) and *Shewanella* sp. (ASV21332) were significantly lower in both the knMyD and the knRel groups compared with the control groups. Our observation suggests that the changes in these bacterial levels were influenced by the impairment of host immunity through knockdown of *PmMyD88* and/or *PmRelish* signaling pathways in *P. monodon*.

**Fig 5:**
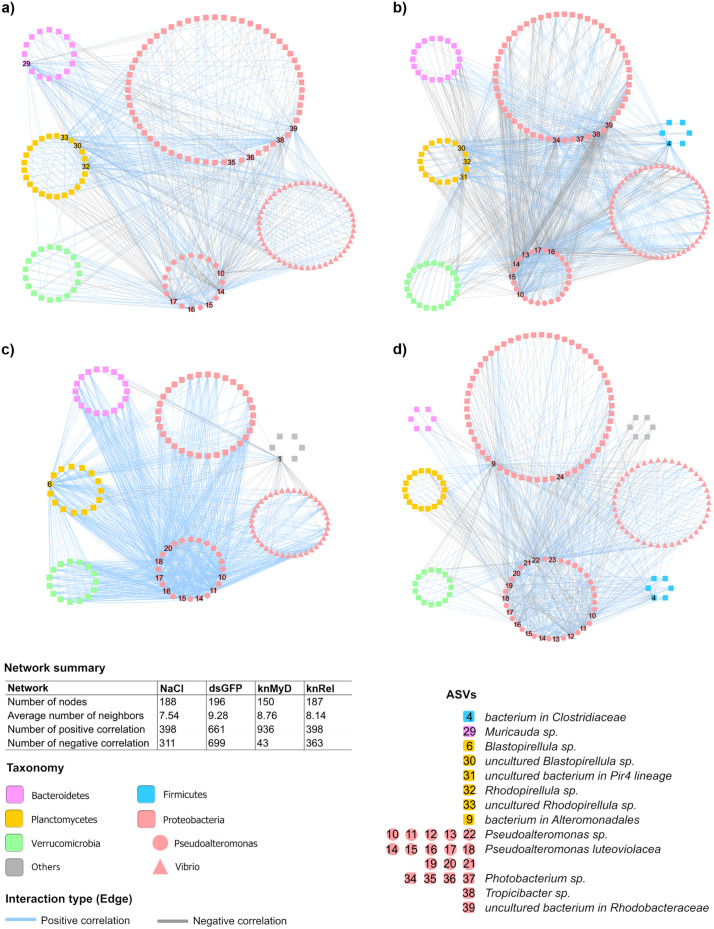
Bacterial co-occurrence networks in intestines of *P. monodon* injected with NaCl, dsGFP, dsMyD88 RNA (knMyD), and dsRelish RNA (knRel) (a, b, c, and d, respectively). Network analysis was performed at the ASV level. Different nodes shapes and colors represent different taxonomic levels. Lines connecting nodes represent correlations (|Spearman correlations coefficient| ≥ 7). Blue lines indicate a positive correlation while grey lines indicate a negative correlation. The number at nodes represents the ASV ID that were significantly different among the groups, as identified by LEfSe.

To determine the effects of immune manipulation of the host shrimp on bacterial interactions, the networking patterns of bacterial communities associated with RNAi knockdown of *PmMyD88* and *PmRelish* pathways were explored using co-occurrence network analysis (Fig 5). The bacterial networks in the shrimp intestine of the NaCl and dsGFP (the control groups) consisted of 188 and 196 nodes, respectively, while in the knMyD and knRel groups (RNAi knockdown of the shrimp immune pathway) had 150 and 187 nodes, respectively. The bacterial networks for all four groups showed similar interaction patterns and taxonomic distribution, in which the networks were dominated by Proteobacteria, Planctomycetes, Bacteroidetes and Verrucomicrobia. *Pseudoalteromonas* was identified as major node in most subnetworks (Fig 5a–5d). *Blastopirellula* (ASV21711) and *Chloroflexi* (ASV5734) were found only in the intestine of knMyD (Fig 5c), whereas a bacterium in *Alteromonadales* (ASV7900) and *Pseudooceanicola* (ASV15214) were found only in intestine of knRel group. Although bacterial diversity could be affected by manipulation of the host immune system using RNAi knockdown, our analysis of the bacterial network showed revealed that host shrimp were able to maintain and remodel bacterial interactions in the intestine environment to satisfy the host and other bacterial members of a community.

### Metabolomics profiles in shrimp intestines under immune gene suppression by RNAi

**Fig 6:**
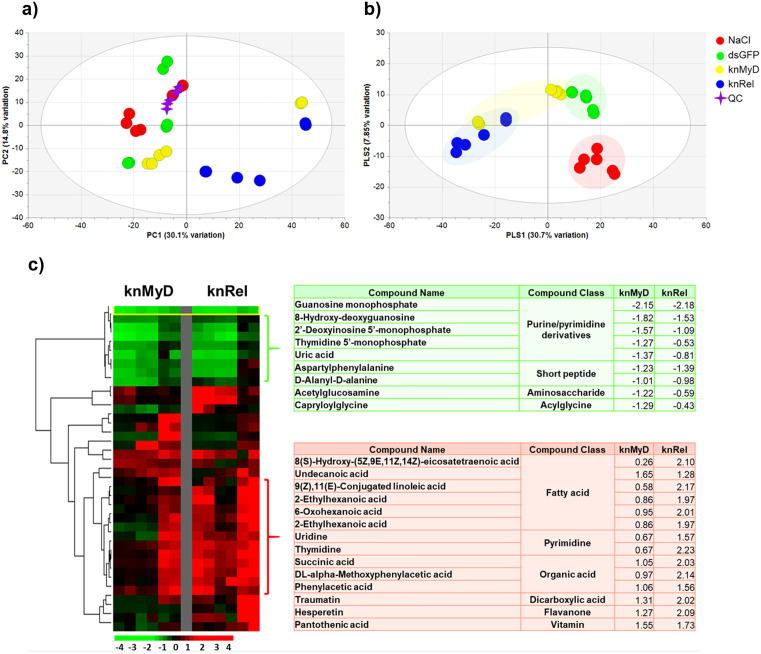
Multivariate statistical analysis and hierarchical clustering of intestinal metabolite profiles following immune pathway knockdown. **(a)** Principal Component Analysis (PCA) and **(b)** Partial Least Squares-Discriminant Analysis (PLS-DA) of the complete metabolite profiles (1,459 features) in shrimp intestines. Samples are grouped by treatment: immune deficiency (IMD) pathway knockdown (yellow), Toll pathway knockdown (blue), compared to the control groups NaCl (red) and dsGFP (green). **(c)** Hierarchical clustering of the subset of intestinal metabolite profiles (131 features) selected based on a Variable Importance in Projection (VIP) score of > 1 and an annotation confidence of > 90%, followed by filtering for a fold change of > 2.0. The right panel shows 9 metabolites that had higher abundance in controls (Control > Treatments) and 14 metabolites that had lower abundance in controls (Control < Treatments).

Metabolite profiles from shrimp intestines under different immune suppressions were analyzed using high-resolution LC-MS-based metabolomics. Four different treatments were compared including i) two different control groups (NaCl and dsGFP treatments) and ii) two different immune suppressions using RNA interference at MyD (knMyD) and at Relish (knRel), which are part of Toll and IMD immune pathways, respectively. A total of 1,459 metabolite features obtained from the negative ESI mode (S3 Table) were subjected to principal component analysis (PCA) to visualize among the sample groups (Fig 6). Unsupervised PCA score plots (PC1 30.1% and PC2 14.8% of total variance) showed that all QC samples (pooled samples) were grouped together, indicating reproducibility of the analysis platform. The PCA revealed that the metabolite profiles of shrimp intestine of NaCl and dsGFP groups were mostly indistinguishable from each other (Fig 6a). The metabolite profile of the shrimp intestines of knRel group was distinct from the control groups, while the metabolites of knMyD group showed high variation among sample replicates. The Supervised Partial Least-Square Discriminant Analysis (PLS-DA, PLS1 30.7% and PLS2 7.8% of the total variance) discriminated among the NaCl, dsGFP and knRel groups, while the profiles of the knMyD group were still inconclusive (Fig 6b). This suggests that the metabolic response of shrimp intestines was associated with knockdown of *PmRelish*, a part of the IMD pathway (knRel), rather than knockdown of *PmMyD88*, which is part of the Toll pathway (knMyD).

A number of 131 out of 1,459 were selected based on variable importance in projection (VIP) greater than 1 to identify metabolites contributing to immune suppressions (Fig 6c). Their relative abundances in the knMyD and knRel groups compared with those from dsGFP group were calculated and clustered according to their alteration patterns. Of these, 23 metabolite features with a change of more than 2-fold and compound annotation confidence greater than 90% were selected. Two major clusters were shown, namely i) decreasing levels of metabolites in both knMyD- and knRel-treated shrimp (9 metabolite features) and ii) increasing levels of metabolites in knRel-treated shrimp (14 metabolite features). The results suggest that suppression of immune system in shrimp perturbed metabolic response in the intestine.

The intestines of both knRel- and knMyD-treated shrimp contained lower levels of purine/pyrimidine derivatives (guanosine monophosphate, 2’-deoxyinosine 5’-monophosphate, 8-hydroxy-deoxyguanosine, thymidine 5’-monophosphate and uric acid), short peptide (D-alanyl-D-alanine, aspartyl phenylalanine), aminosaccharide (acetylglucosamine) and acyl glycine (capryloylglycine). In contrast, the intestines of the knRel group showed higher levels of vitamins (pantothenic acid, vitamin B5), fatty acids (6-oxohexanoic acid, 2-ethylhexanoic acid, 9(Z),11(E)-conjugated linoleic acid, 8(S)-hydroxy-(5Z,9E,11Z,14Z)-eicosatetraenoic acid (8-HETE), and undecanoic acid), organic acid (DL-alpha-methoxyphenylacetic acid, succinic acid, phenylacetic acid, D-alpha-hydroxyglutaric acid), dicarboxylic acid (traumatin), flavanone (hesperetin) and pyrimidine (uridine and thymidine) (Fig 6c).

### Alterations in host immune expression and intestinal dysbiosis following *Pm*Relish knockdown

**Fig 7:**
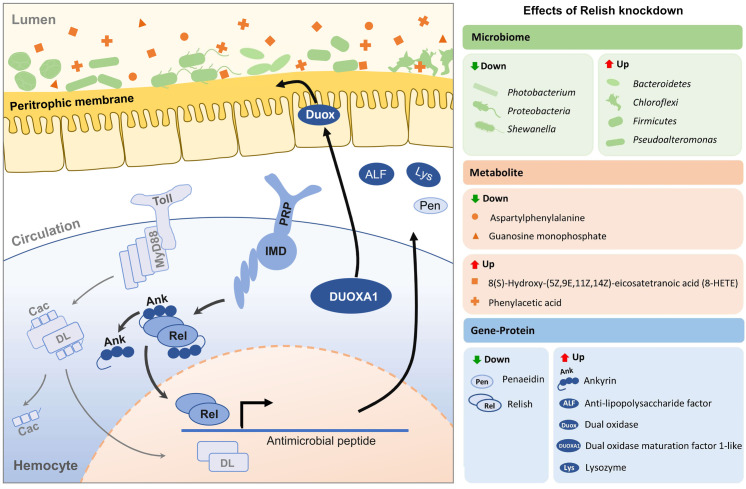
Proposed model of the IMD-mediated host-microbiome-metabolome interactions following *Relish* knockdown in the black tiger shrimp, *Penaeus monodon.* The schematic illustrates the downstream effects of *Pm*Relish suppression on hemocyte immune signaling, gut microbiota composition, and metabolic profiles. (Left) In the hemocyte, the IMD pathway signaling cascade involves the interactions of PRP and IMD, leading to the translocation of Relish (Rel). *Pm*Relish knockdown (knRel) disrupts the transcription of specific antimicrobial peptides (AMPs). This disruption leads to a compensatory feedback loop involving the upregulation of the *Duox/DUOXA1* complex at the peritrophic membrane. (Right) Summary of differential expression analysis. *Pm*Relish knockdown results in the downregulation of *penaeidin* and specific suppression of *Relish*, alongside a compensatory upregulation of *ankyrin* (*Ank*), Anti-lipopolysaccharide factor (*ALF*), and *lysozyme* (*Lys*). This immune modulation is associated with significant dysbiosis in the gut lumen, characterized by a reduction in Proteobacteria (specifically *Photobacterium* and *Shewanella*) and an enrichment of Bacteroidetes, Chloroflexi, Firmicutes, and *Pseudoalteromonas*. These microbial shifts coincide with metabolic alterations, specifically the depletion of aspartylphenylalanine and guanosine monophosphate, and the accumulation of phenylacetic acid and the eicosanoid 8-HETE.

To elucidate the role of the IMD pathway in maintaining gut homeostasis, we constructed a multi-omics interaction model (Fig 7). While both Toll and IMD pathways typically regulate innate immune responses [50], our findings revealed that the knockdown of *PmRelish* (IMD pathway) exerted a disproportionately greater impact on the gut ecosystem compared to *PmMyD88* (Toll pathway). The suppression of *Pm*Relish significantly altered the transcriptional landscape of immune effectors. While the antimicrobial peptide *penaeidin* was downregulated concomitant with *Relish* suppression, we observed a distinct compensatory upregulation of other immune-related genes, including *ankyrin*, *anti-lipopolysaccharide factor* (ALF), *lysozyme*, and the reactive oxygen species-producing complex *Duox*/*DUOXA1*. These immunological shifts were mirrored by significant alterations in the gut microbiome structure. The knockdown of Relish induced a state of dysbiosis characterized by a depletion of proteobacterial members, specifically *Photobacterium* and *Shewanella*. Conversely, the knockdown led to the proliferation of *Bacteroidetes*, *Chloroflexi*, *Firmicutes*, and *Pseudoalteromonas*. Furthermore, metabolomic profiling revealed that this host-microbiome shift resulted in a distinct metabolic signature. The luminal microenvironment showed elevated levels of phenylacetic acid and the inflammatory mediator 8(S)-Hydroxy-(5Z,9E,11Z,14Z)-eicosatetraenoic acid (8-HETE). In contrast, aspartylphenylalanine and guanosine monophosphate levels were significantly reduced, suggesting an alteration in amino acid and nucleotide metabolism linked to the shifted microbial community.

## Discussion

While the intricate relationship between host innate immunity and the gut microbiota is well-studied in model organisms such as *Drosophila*, its mechanisms in commercially important invertebrates like the black tiger shrimp, *P. monodon*, are less understood. Our study employed an integrated multi-omics approach to dissect this relationship by systematically suppressing two canonical innate immune pathways, Toll and IMD, via RNAi-mediated knockdown of MyD88 and Relish, respectively. Perturbing the immune system at under non-pathogenic, baseline conditions, allowed us to understand its fundamental role in structuring the commensal microbial community and maintaining gut homeostasis. Our findings showed that under these conditions, the IMD pathway involved as the primary regulator of gut homeostasis in the black tiger shrimp.

The disproportionately larger transcriptomic response to *PmRelish* knockdown (1,362 differentially expressed transcripts) compared to *PmMyD88* suppression (333 transcripts) suggests the IMD pathway is a more influential regulator of basal immune and physiological gene expression. This finding is consistent with its known role in responding to Gram-negative bacteria [51], which are dominant members of the shrimp gut microbiota, including genera like *Vibrio*, *Pseudoalteromonas* and *Photobacterium* [45,52,53]. Our findings are consistent with studies in other invertebrates such as *Drosophila* which show the IMD pathway is a key regulator of homeostatic immunity, and actively managing commensal bacterial populations to maintain gut homeostasis [24,54]. Moreover, the suppression of the shrimp immune system, IMD and Toll pathways, also showed observable functional consequences, as evidenced by a decrease in the total hemocyte count (THC), affecting all three hemocyte major classes (hyaline, granular, and semi-granular cells). Total hemocyte is a key indicator of cellular immune health in shrimp, as these cells are central to the host’s defense mechanisms against pathogens and other stressors (Cerenius and Söderhäll, 2012; Rodríguez and Le Moullac, 2000).

We observed differential gene expression patterns in the host transcriptome, including a selective response in antimicrobial peptides (AMPs). In the knRel group, there was a significant upregulation of transcripts for *lysozyme C-like* and *anti-lipopolysaccharide factor (ALF)*, while transcripts for *penaeidins* were strongly suppressed. This targeted alteration in AMP expression showed that the IMD pathway, through the transcription factor, *Relish*, actively shaped the gut’s microbial community. In parallel, these transcriptomic changes were correlated to significant shifts in the gut microbiota. Although the overall microbial community structure remained stable at the phylum level, with a dominance of Proteobacteria, Bacteroidetes, Verrucomicrobia, and Planctomycetes, the significant shifts were observable at the genus level. Our microbiome analysis revealed that the abundance of common Gram-negative genera in shrimp gut [52,55], such as *Photobacterium* and *Shewanella*, was significantly reduced in both knMyD and knRel groups compared to controls. While other Gram-negative taxa declined, LEfSe analysis indicated that specific *Pseudoalteromonas* ASVs were significantly enriched following *Relish* suppression. The distinct response among Gram-negative bacteria could be explained by the selective modulation of antimicrobial peptides (AMPs) observed in the transcriptomic profiling. The knockdown of *Relish* led to a strong suppression of *penaeidin 5* (log_2_ fold change = −3.62) and *stylicin 1*, both of which are IMD-dependent AMPs, but simultaneously triggered a compensatory upregulation of *lysozyme C-like* (log_2_ fold change = 2.44) and *anti-lipopolysaccharide factors (ALFs)*. Notably, ALFs and lysozymes are typically induced through the Toll pathway [56], supporting the interpretation that Toll-mediated AMPs were activated to compensate for the loss of IMD-mediated effectors. Therefore, the suppression of *Relish*, a primary effector of the IMD pathway, did not simply shut down all AMP production, but instead triggered a compensatory Relish-independent immune response and selective pressure on the microbial community. Furthermore, our observation suggests that *lysozyme C* and *ALFs* could effectively regulate susceptible genera like *Photobacterium*. In contrast, the enriched *Pseudoalteromonas* species likely possess outer membrane modifications or resistance mechanism allowing them to tolerate this altered immune environment, enabling them to utilize resources made available by the reduction of other commensal bacteria. Thus, the IMD pathway appears to fine-tune community composition through specific regulation of antimicrobial effectors rather indiscriminately suppressing all Gram-negative bacteria. Furthermore, several members of Firmicutes, Bacteroidetes, and Planctomycetes were enriched specifically in the knRel group, suggesting these taxa were early responders to the compromised host immunity, potentially key players in the host-microbe crosstalk.

Beyond antimicrobial activity, we also observed metabolic shifts linking immunity to homeostasis. There was a significant decrease in metabolites associated with bacterial structures, such as of D-alanyl-D-alanine (a key component of bacterial peptidoglycan [57]) and N-acetylglucosamine (a component of both bacterial cell walls [58] and the host intestinal mucous layer [59]). This supports the observation of bacterial disturbance and a potential weakening of the protective peritrophic gut barrier of shrimp. Furthermore, the accumulation of specific metabolites in the knRel group points to microbial dysbiosis and a potential pro-inflammatory state in the host shrimp. The intestines of knRel-treated shrimp showed an accumulation of host-derived lipids and inflammatory markers, including various fatty acids and 8-HETE. The elevation of these lipid metabolites suggests a disruption in the crosstalk between IMD signaling and metabolic regulation. In *Drosophila*, the IMD pathway interacts with metabolic sensors such as lipid metabolic pathways to balance immunity with energy homeostasis [60]. Consequently, the increase in free fatty acids and 8-HETE in our Relish-suppressed shrimp may reflect a compensatory mobilization of lipids for immune signaling (eicosanoids) or repair mechanisms in the absence of IMD regulation. This provides an evidence that disrupting IMD pathway homeostasis can trigger a metabolic shift toward a pro-inflammatory state characterized by lipid dysregulation.

The compromised state and the weakening of the gut barrier likely led to a host-driven compensatory responses, as we observed a significant upregulation of the *dual oxidase maturation factor 1-like* (*DUOXA1*) transcript in the hemocytes of the knRel group (log_2_ fold change = 7.56). In other invertebrates, dual oxidase (DUOX) plays a crucial role in maintaining the structural integrity of the gut’s physical barriers, such as the peritrophic matrix (PM). For instance, in the oriental fruit fly, *Bactrocera dorsalis*, Duox-derived reactive oxygen species (ROS) are crucial for PM formation and stability [61]. Importantly, a compromised PM, whether due to a lack of DUOX activity or direct damage, leads to increased gut permeability and heightened immune reactivity of the IMD pathway [62]. Therefore, the highly induced expression of DUOXA1 in our study could represent a host’s molecular mechanism to reinforce the gut’s physical barrier, likely to compensate for the disrupted immune signaling and the observed shifts in the gut microbiota and metabolic profiles. The observed metabolic changes also suggested a state of microbial dysbiosis. The elevated levels of undigested plant-based dietary compounds (e.g., traumatin, hesperetin) indicate that the imbalanced microbiota may have compromised the host’s ability to process nutrients, potentially impacting its nutritional status. This is also consistent with the accumulation of pro-inflammatory metabolites such as succinic acid and 8-HETE, which suggests that the disruption of the IMD pathway may have led to an underlying state of gut inflammation.

In conclusion, our study provides a comprehensive, multi-omics view of the homeostatic interplay between the innate immune system and the gut microbiota in *P. monodon*. We propose a model where the innate immune IMD pathway acts as a key regulator of this balance. As illustrated in our findings (Fig 7), the IMD pathway, through the transcription factor Relish, regulates the expression of an array of antimicrobial peptides in hemocytes that shape the gut’s microbial community. Suppressing this pathway could perturb the shrimp gut ecosystem, leading to a decrease in key bacterial taxa like *Photobacterium* and an altered metabolic environment characterized by the accumulation of pro-inflammatory markers such as 8-HETE. We hypothesize that the host attempts to compensate for this barrier dysfunction by upregulating dual oxidase (DUOX), a key enzyme in fortifying the gut’s physical defenses. This work reveals, for the first time in a commercially vital crustacean, that the IMD pathway is not just a defense mechanism against acute infection, but a fundamental regulator of commensal microbiota and metabolic homeostasis. These findings identify a promising biological target for improving shrimp aquaculture, suggesting that strategies aimed at supporting this immune axis or maintaining microbiome resilience could significantly enhance shrimp health and the sustainability of the industry.

## Supporting information

S1 Fig Venn diagram shows the numbers of up- and down- regulated DEGs between knockdown of *PmMyD88* (knMyd88) and *PmRelish* (knRel) *P. monodon* groups (compare with dsGFP injection). (DOCX)

S2 Fig Validation of RNAseq data by quantitative real-time PCR of immune-related genes that show differential expression in KnRelish and KnMyD88 condition. (DOCX)

S3 Fig Rarefaction cureve analysis for 16S amplicon sequences obtained from shrimp intestines in knockdown of *PmMyD88* (knMyd88) and *PmRelish* (knRel) *P. monodon* groups. (DOCX)

S4 Fig Microbial diversity and community structure in intestine and water samples in different treatments (NaCl, dsGFP, knMyD, and knRel). (A) Shannon index for samples collected from the intestine and water. Samples were treated with NaCl, dsGFP, knMyD, and knRel. The table below summarizes the result of ANOVA analysis. (B) Principal coordinate analysis of weighted UniFrac distance (PCoA) plots demonstrating the beta diversity of bacterial communities. The table below summarizes the result of PERMANOVA analysis.
(DOCX)

S1 Table Primers used in quantitative real-time PCR analysis. (DOCX)

S2 Table List of genes showing differential expression between knMyD and dsGFP, and knRel and dsGFP. The significant differentially expression were determined by a greater than two-fold change (|log_2_ fold change| ≥ 1) and p-value < 0.05.
(XLSX)

S3 Table Metabolite profiles from shrimp intestine under suppression of immune pathways using RNAi. A total of 1,459 metabolite features acquired by negative ESI mode using LC-HRMS/MS were Pareto scaled.
(XLSX)
