# Supplementary material for: The innate immune IMD pathway is a key regulator of gut microbiome and metabolic homeostasis in the black tiger shrimp (Penaeus monodon)
Source: PLoS One. 2025 Dec 16;20(12):e0338796. doi: 10.1371/journal.pone.0338796 (PMC12707661; doi:10.1371/journal.pone.0338796)

**S1 Figure.** Venn diagram shows the numbers of up- and down- regulated DEGs between knockdown of *PmMyD88* (knMyd88) and *PmRelish* (knRel) *P. monodon* groups (compare with dsGFP injection).


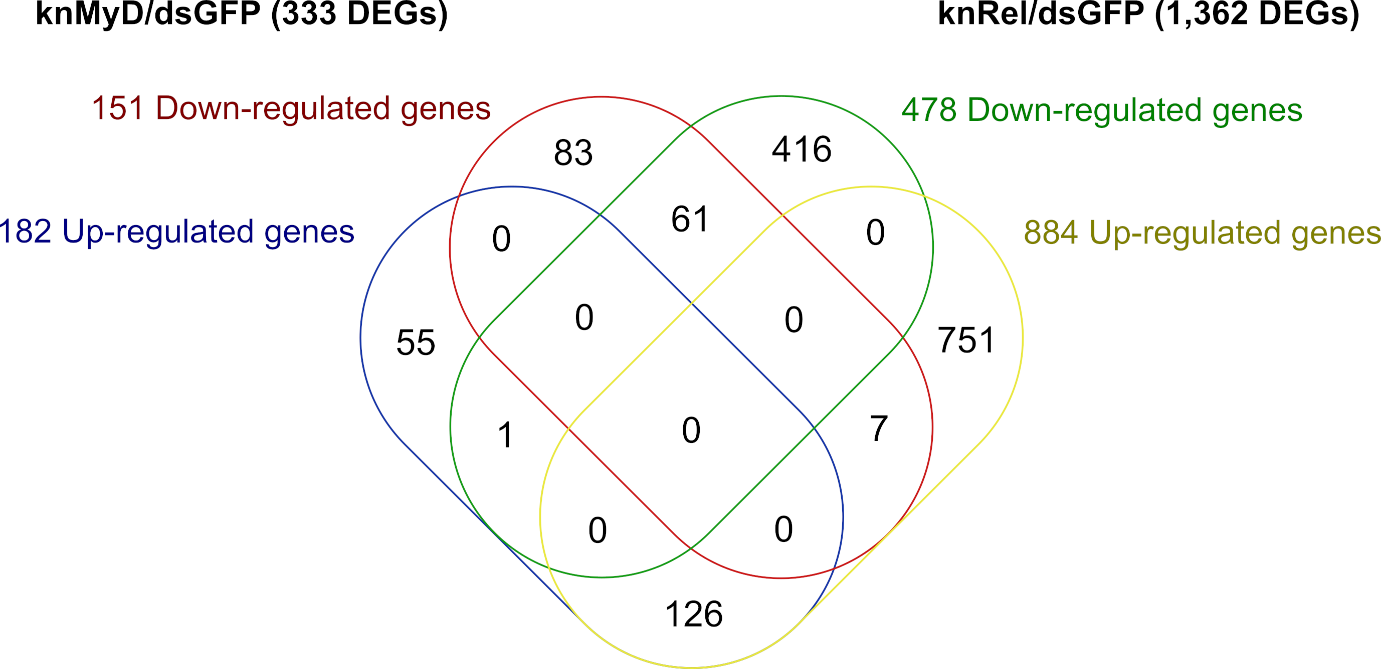

Supplement: S1 Fig — (DOCX) [file pone.0338796.s001.docx]
