# Supplementary material for: The innate immune IMD pathway is a key regulator of gut microbiome and metabolic homeostasis in the black tiger shrimp (Penaeus monodon)
Source: PLoS One. 2025 Dec 16;20(12):e0338796. doi: 10.1371/journal.pone.0338796 (PMC12707661; doi:10.1371/journal.pone.0338796)

**S2 Figure.** Validation of RNAseq data by quantitative real-time PCR of immune-related genes that show differential expression in KnRelish and KnMyD88 condition.


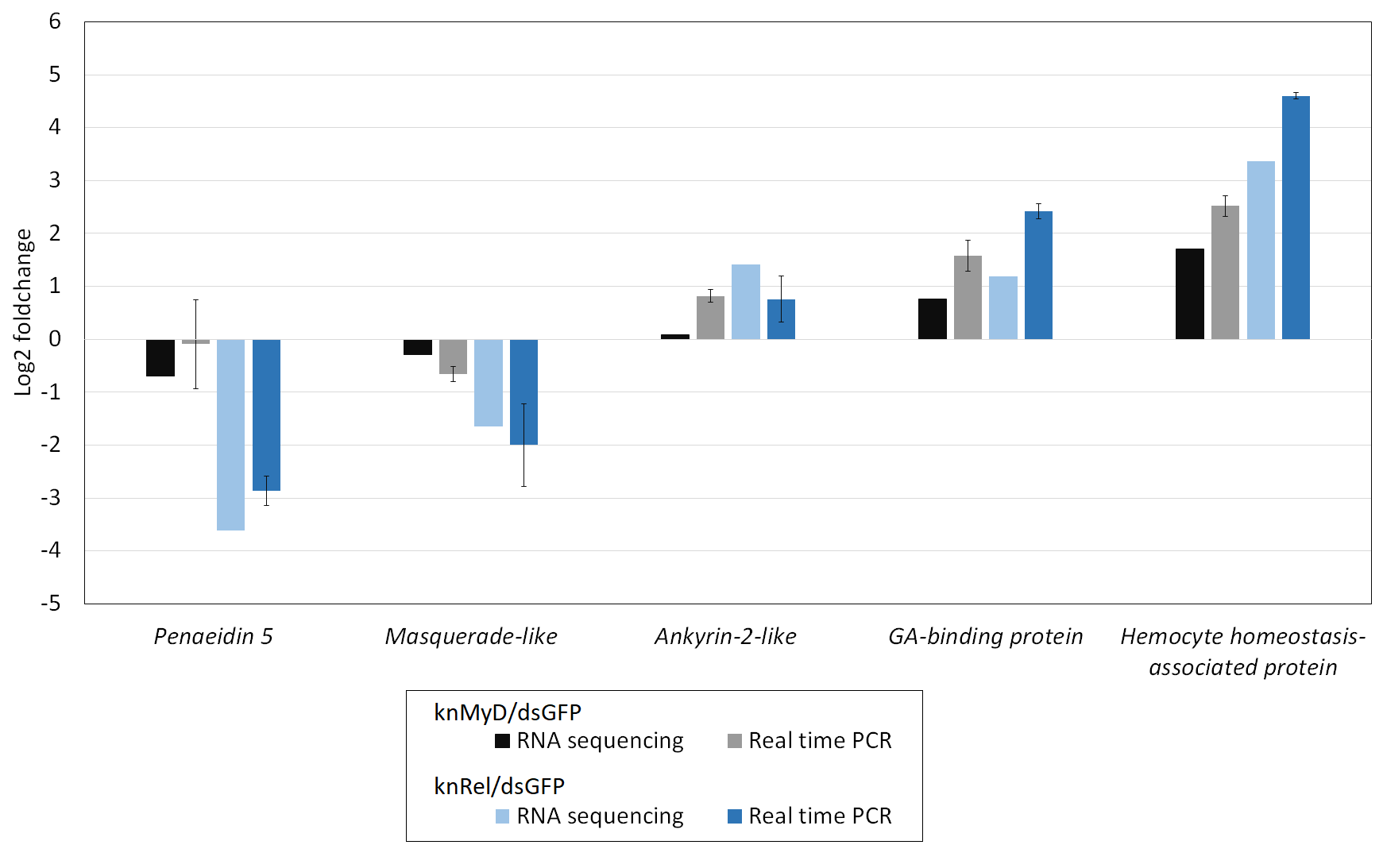

Supplement: S2 Fig — (DOCX) [file pone.0338796.s002.docx]
