# Supplementary material for: The innate immune IMD pathway is a key regulator of gut microbiome and metabolic homeostasis in the black tiger shrimp (Penaeus monodon)
Source: PLoS One. 2025 Dec 16;20(12):e0338796. doi: 10.1371/journal.pone.0338796 (PMC12707661; doi:10.1371/journal.pone.0338796)

**S3 Figure.** Rarefaction cureve analysis for 16S amplicon sequences obtained from shrimp intestines in knockdown of *PmMyD88* (knMyd88) and *PmRelish* (knRel) *P. monodon* groups.


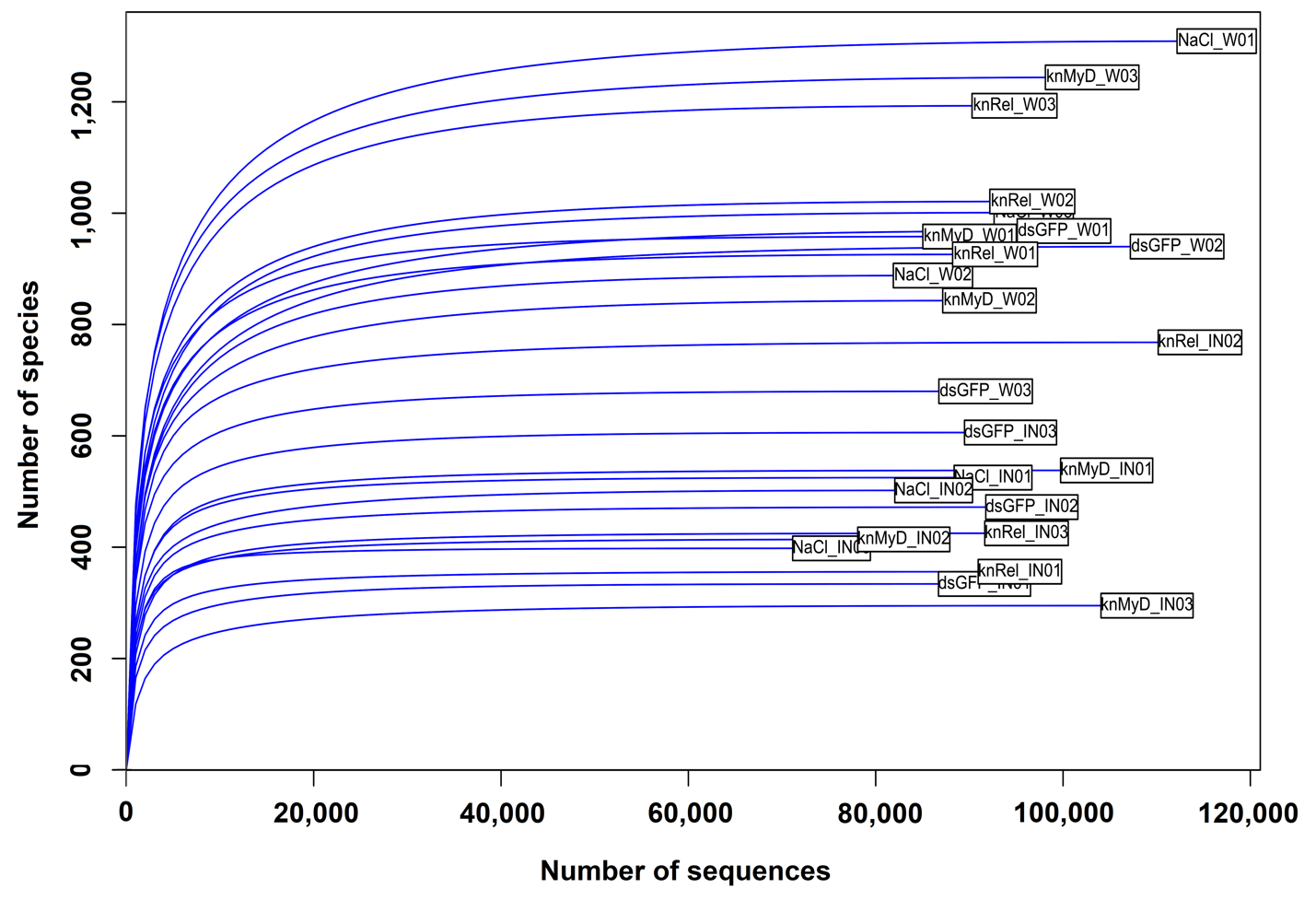

Supplement: S3 Fig — (DOCX) [file pone.0338796.s003.docx]
