# Supplementary material for: The innate immune IMD pathway is a key regulator of gut microbiome and metabolic homeostasis in the black tiger shrimp (Penaeus monodon)
Source: PLoS One. 2025 Dec 16;20(12):e0338796. doi: 10.1371/journal.pone.0338796 (PMC12707661; doi:10.1371/journal.pone.0338796)

**S4 Figure.** Microbial diversity and community structure in intestine and water samples in different treatments (NaCl, dsGFP, knMyD, and knRel). (A) Shannon index for samples collected from the intestine and water. Samples were treated with NaCl, dsGFP, knMyD, and knRel. The table below summarizes the result of ANOVA analysis. (B) Principal coordinate analysis of weighted UniFrac distance (PCoA) plots demonstrating the beta diversity of bacterial communities. The table below summarizes the result of PERMANOVA analysis.


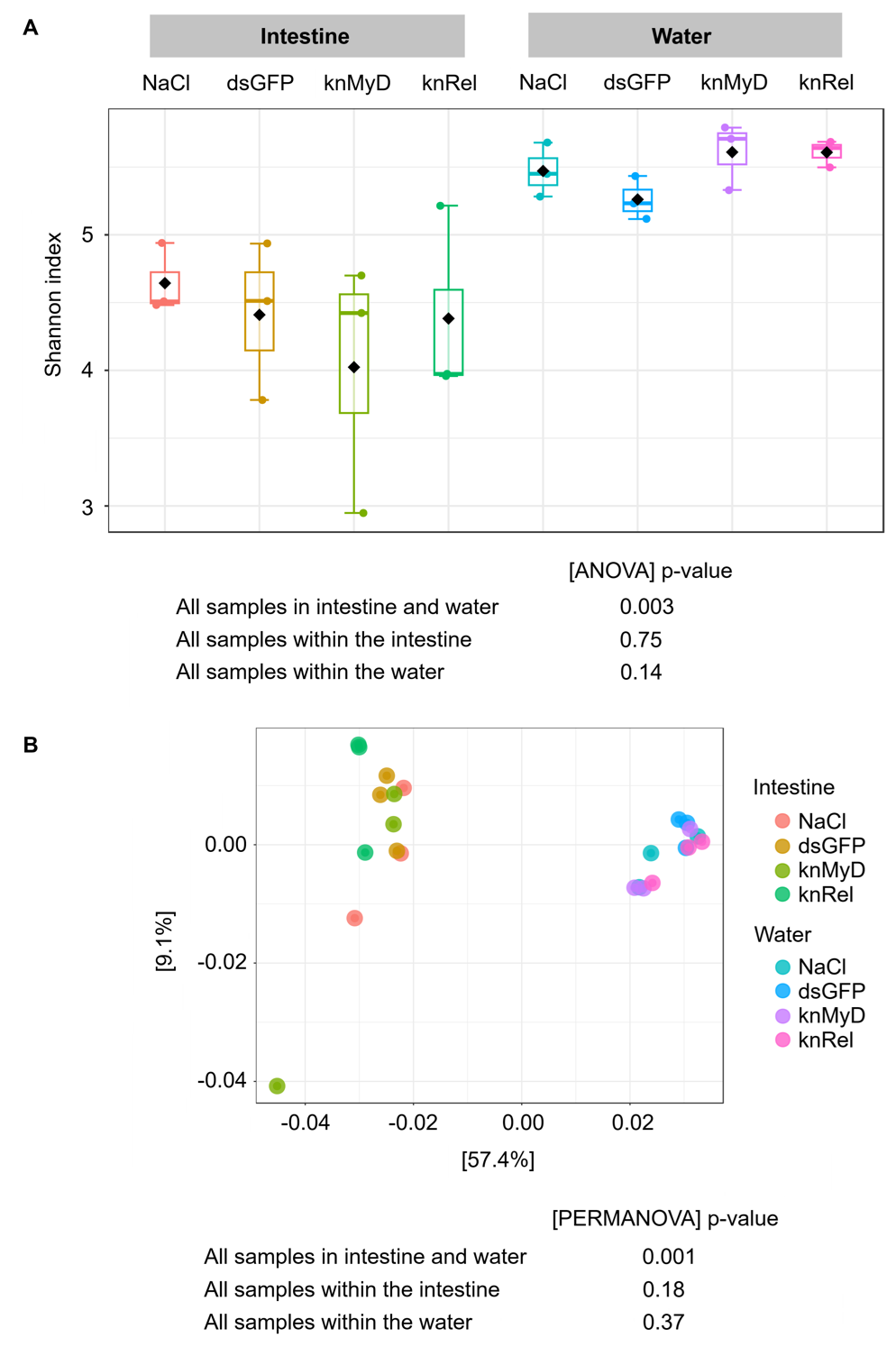

Supplement: S4 Fig — (A) Shannon index for samples collected from the intestine and water. Samples were treated with NaCl, dsGFP, knMyD, and knRel. The table below summarizes the result of ANOVA analysis. (B) Principal coordinate analysis of weighted UniFrac distance (PCoA) plots demonstrating the beta diversity of bacterial communities. The table below summarizes the result of PERMANOVA analysis. (DOCX) [file pone.0338796.s004.docx]
