# Supplementary material for: The innate immune IMD pathway is a key regulator of gut microbiome and metabolic homeostasis in the black tiger shrimp (Penaeus monodon)
Source: PLoS One. 2025 Dec 16;20(12):e0338796. doi: 10.1371/journal.pone.0338796 (PMC12707661; doi:10.1371/journal.pone.0338796)
Supplement: S1 Table — (DOCX) [file pone.0338796.s005.docx]

**S1 Table.** Primers used in quantitative real-time PCR analysis

| **Gene** | **Primer sequence (5' to 3')** | **Product size (bp)** |
| --- | --- | --- |
| *Pm*MyD88 | F: GTGCACCAGAGTCATTGTAG  R: GGGAGTGGCAGAAACTTATC | - |
| *Pm*Relish | F: TCTCCAGGTGAGCACTCAGTTGGC  R: GCTGTAGCTGTTGCTGTTGTTGAG | - |
| *Penaeidin 5* | F: ACTCCAGACCACCCTATGGA | 122 |
|  | R: GAACTGTCTGCAGCAAGCAA |  |
| *Masquerade-like* | F: CATCAACAGCGTTTGTGTCC | 141 |
|  | R: TAGTTGTCCACCAGGGGAAG |  |
| *Ankyrin-2-like* | F: GCTCCTCGAGTTTCATCTGC | 143 |
|  | R: TGCTTCTCGACTCCAATTCC |  |
| *GA-binding protein nkyrin-I8* | F: ACACGTGTGACACCCACACT | 110 |
|  | R: AGTCGATCCCTTACGCAGAA |  |
| *Hemocyte homeostasis-associated protein* | F: TTCGAGTGTGGAAAATGCAG | 118 |
|  | R: TCTCGTGCTTGACCTGTTTG |  |
| *Elongation factor 1a* | F: CAGGCGTACTGGTAAGGAACTGG | 123 |
|  | R: AGAGGAGCATACTGTTGGAAGGTCTC |  |
